# Surface environment complication makes Ag_29_ nanoclusters more robust and leads to their unique packing in the supracrystal lattice†

**DOI:** 10.1039/d1sc06002c

**Published:** 2022-01-03

**Authors:** Chao Xu, Qianqin Yuan, Xiao Wei, Hao Li, Honglei Shen, Xi Kang, Manzhou Zhu

**Affiliations:** Department of Chemistry, Centre for Atomic Engineering of Advanced Materials, Anhui Province Key Laboratory of Chemistry for Inorganic/Organic Hybrid Functionalized Materials, Anhui University Hefei 230601 P. R. China kangxi_chem@ahu.edu.cn; Key Laboratory of Structure and Functional Regulation of Hybrid Materials, Anhui University, Ministry of Education Hefei 230601 P. R. China zmz@ahu.edu.cn

## Abstract

Silver nanoclusters have received unprecedented attention in cluster science owing to their promising functionalities and intriguing physical/chemical properties. However, essential instability significantly impedes their extensive applications. We herein propose a strategy termed “surface environment complication” to endow Ag_29_ nanoclusters with high robustness. The Ag_29_(S-Adm)_18_(PPh_3_)_4_ nanocluster with monodentate PPh_3_ ligands was extremely unstable and uncrystallizable. By substituting PPh_3_ with bidentate PPh_2_py with dual coordination sites (*i.e.*, P and N), the Ag_29_ cluster framework was twisted because of the generation of N–Ag interactions, and three NO_3_ ligands were further anchored onto the nanocluster surface, yielding a new Ag_29_(S-Adm)_15_(NO_3_)_3_(PPh_2_py)_4_ nanocluster with high stability. The metal-control or ligand-control effects on stabilizing the Ag_29_ nanocluster were further evaluated. Besides, Ag_29_(S-Adm)_15_(NO_3_)_3_(PPh_2_py)_4_ followed a unique packing mode in the supracrystal lattice with several intercluster channels, which has yet been observed in other M_29_ cluster crystals. Overall, this work presents a new approach (*i.e.*, surface environment complication) for tailoring the surface environment and improving the stability of metal nanoclusters.

## Abstract 2

A strategy of “surface environment complication” has been exploited to endow Ag_29_ nanoclusters with high robustness and a unique packing mode in the supracrystal lattice.

## Graphical abstract

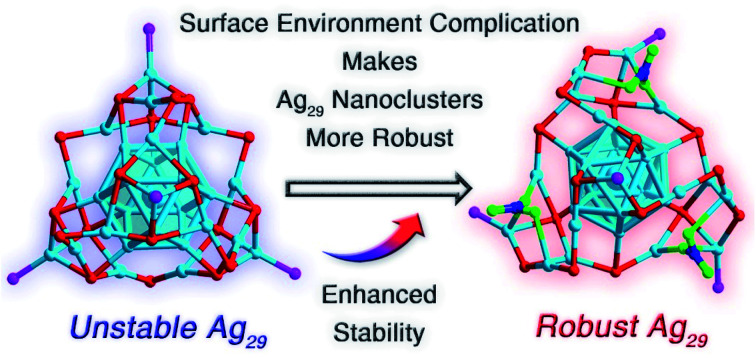

## 1 Introduction

Since the advent of metal nanoclusters with atomic precision, these novel nanomaterials have garnered significant interest because of their accurate compositions/constructions and intriguing physicochemical properties.^1–8^ Indeed, owing to their quantum size effect and discrete electronic energy levels, metal nanoclusters and cluster-based nanomaterials display atomic structure tunable properties, that is, slight tailoring of structures of nanoclusters can trigger remarkable differences in their performances.^9–13^ Besides, metal nanoclusters have been used as ideal platforms for the meticulous investigation of structure–property correlations.^14–20^ Consequently, metal nanoclusters are an emerging class of programmable nanomaterials for several promising applications, such as catalysis, drug delivery, energy storage, and biological applications.^21–24^

In the past two decades, silver nanoclusters have received unprecedented attention in cluster science.^25–40^ It is widely accepted that silver nanoclusters exhibit promising functionalities and intriguing physical/chemical properties that are obviously different from their gold counterparts.^27^ Ag-based metal nanoclusters generally display strong photoluminescence that renders them optically active nanomaterials for sensors or biological applications.^41–43^ However, Ag nanoclusters are essentially unstable relative to Au nanoclusters, which significantly impedes their extensive applications. Developing new approaches to enhance the nanocluster stability remains highly desired.

Recently, our group has developed a M_29_(S-Adm)_18_(PPh_3_)_4_ (where S-Adm is 1-adamantanethiol) nanocluster system for mapping the structure–property correlations at the atomic level.^44–46^ Although several M_29_ nanoclusters, *e.g.*, Pt_1_Ag_28_(S-Adm)_18_(PPh_3_)_4_ (Pt_1_Ag_28_-PPh_3_ for short), Au_1_Ag_28_(S-Adm)_18_(PPh_3_)_4_, and Pt_1_Ag_12_Cu_16_(S-Adm)_18_(PPh_3_)_4_, have been controllably synthesized and structurally determined, the homo-metal Ag_29_(S-Adm)_18_(PPh_3_)_4_ (Ag_29_-PPh_3_ for short) nanocluster was extremely unstable and uncrystallizable.^46^ We remain committed to stabilizing the homo-silver Ag_29_ nanocluster with a new approach.

Herein, a “surface environment complication” strategy has been exploited to endow the Ag_29_ nanocluster with high robustness. By substituting the monodentate PPh_3_ (with only the P coordination site) in previously reported Ag_29_-PPh_3_ with bidentate PPh_2_py (with P and N dual coordination sites), the nanocluster surface structure underwent a twist due to the generation of N–Ag interactions. Besides, three NO_3_ ligands were further anchored onto the nanocluster surface, making the metallic kernel entirely wrapped. The obtained Ag_29_(S-Adm)_15_(NO_3_)_3_(PPh_2_py)_4_ (Ag_29_-PPh_2_py for short) nanocluster was much more robust relative to Ag_29_-PPh_3_, and its structure was successfully determined by single-crystal X-ray diffraction. Furthermore, based on this nanocluster template, the metal-control and ligand-control effects on stabilizing the Ag_29_ framework were evaluated. Moreover, at the supramolecular level, Ag_29_-PPh_2_py followed a unique packing mode in the crystal lattice with several intercluster channels, while such an aggregation pattern has yet been discovered in other M_29_ cluster crystals.

## 2 Experimental methods

### Materials

All the following reagents were purchased from Sigma-Aldrich and used without further purification: silver nitrate (AgNO_3_, 99.5%, metal basis), hexachloroplatinic(iv) acid (H_2_PtCl_6_·6H_2_O, 99.9% metals basis), 1-adamantanethiol (Adm-SH, C_10_H_15_SH, 99%), triphenylphosphine (PPh_3_, 99%), diphenyl-2-pyridylphosphine (PPh_2_py, 97%), sodium borohydride (NaBH_4_, 99%), methylene chloride (CH_2_Cl_2_, HPLC grade), methanol (CH_3_OH, HPLC grade), ethanol (CH_3_CH_2_OH, HPLC grade), and *n*-hexane (C_6_H_12_, HPLC grade).

### Synthesis of Ag_29_(S-Adm)_18_(PPh_3_)_4_ (Ag_29_-PPh_3_)

The preparation of Ag_29_-PPh_3_ was based on a reported method.^46^

### Synthesis of Pt_1_Ag_28_(S-Adm)_18_(PPh_3_)_4_ (Pt_1_Ag_28_-PPh_3_)

The preparation of Pt_1_Ag_28_-PPh_3_ was based on a reported method.^46^

### Preparation of Ag_29_(S-Adm)_15_(NO_3_)_3_(PPh_2_py)_4_ (Ag_29_-PPh_2_py)

In a 50 mL round-bottom flask, 94 mg of AgNO_3_ was dissolved in 5 mL of MeOH and 10 mL of EtOH, and 50 mg of Adm-SH was added under vigorous stirring. After 20 min, 100 mg of PPh_2_py was added. Shortly after this, 10 mg of NaBH_4_ (dissolved in 1 mL of EtOH) was poured in, and the reaction was continued for 12 hours. The obtained solution was centrifuged at 10 000 rpm for 5 minutes, and then the supernatant was collected and evaporated to get the dry product, which was then washed several times with *n*-hexane to get the final product, *i.e.*, Ag_29_-PPh_2_py. The yield was about 30% based on the Ag element (calculated from AgNO_3_).

### Preparation of Pt_1_Ag_28_(S-Adm)_18_(PPh_2_py)_4_ (Pt_1_Ag_28_-PPh_2_py)

94 mg of AgNO_3_ used to synthesize Ag_29_-PPh_2_py was substituted by 94 mg of AgNO_3_ and 10 mg of H_2_PtCl_6_·6H_2_O. Other conditions remained unchanged. The yield for the synthesis of Pt_1_Ag_28_-PPh_2_py was about 45% based on the Ag element (calculated from AgNO_3_).

### Crystallization of the Ag_29_ nanocluster series

Single crystals of Ag_29_-PPh_2_py or Pt_1_Ag_28_-PPh_2_py were cultivated at −4 °C by liquid-diffusing *n*-hexane into the CH_2_Cl_2_ solution of each nanocluster. After a week, red crystals were collected, and the structures of these nanoclusters were determined. Of note, in order to accelerate the crystallization process and improve the crystal quality, the counterions (*i.e.*, Cl^−^) in these nanoclusters were replaced by SbF_6_^−^ or BPh_4_^−^.^47^ The reaction equation was [Ag_29_(S-Adm)_15_(NO_3_)_3_(PPh_2_py)_4_]Cl_3_ + 3SbF_6_^−^ → [Ag_29_(S-Adm)_15_(NO_3_)_3_(PPh_2_py)_4_](SbF_6_)_3_ + 3Cl^−^ or [Pt_1_Ag_28_(S-Adm)_18_(PPh_2_py)_4_]Cl_2_ + 2BPh_4_^−^ → [Pt_1_Ag_28_(S-Adm)_18_(PPh_2_py)_4_](BPh_4_)_2_ + 2Cl^−^.

### Characterization

The optical absorption spectra of nanoclusters were recorded using an Agilent 8453 diode array spectrometer.

Electrospray ionization mass spectrometry (ESI-MS) measurements were performed by using a Waters XEVO G2-XS QTof mass spectrometer. The sample was directly infused into the chamber at 5 μL min^−1^. For preparing the ESI samples, nanoclusters were dissolved in CH_2_Cl_2_ (1 mg mL^−1^) and diluted (v/v = 1 : 1) with CH_3_OH.

Infrared (IR) measurements were recorded on a Bruker Vertex 80sv Fourier transform IR spectrometer.

### X-ray crystallography

The data collection for single-crystal X-ray diffraction (SC-XRD) of Ag_29_-PPh_2_py was carried out on a Bruker Smart APEX II CCD diffractometer under a nitrogen flow, using graphite-monochromatized Mo Kα radiation (*λ* = 0.71073 Å). The data collection for single-crystal X-ray diffraction (SC-XRD) of Pt_1_Ag_28_-PPh_2_py was carried out on a Stoe Stadivari diffractometer under a nitrogen flow, using graphite-monochromatized Cu Kα radiation (*λ* = 1.54186 Å). Data reductions and absorption corrections were performed using the SAINT and SADABS programs, respectively. The structure was solved by direct methods and refined with full-matrix least squares on *F*^2^ using the SHELXTL software package. All non-hydrogen atoms were refined anisotropically, and all the hydrogen atoms were set in geometrically calculated positions and refined isotropically using a riding model. All crystal structures were treated with PLATON SQUEEZE. The diffuse electron densities from these residual solvent molecules were removed. The CCDC number of the Ag_29_-PPh_2_py nanocluster is 2115749. The CCDC number of the Pt_1_Ag_28_-PPh_2_py nanocluster is 2117814.

## 3 Results and discussion

**Fig. 1:**
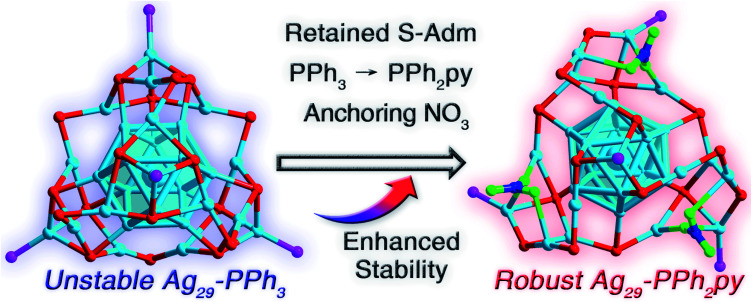
Structural comparison between unstable Ag_29_-PPh_3_ and robust Ag_29_-PPh_2_py. Compared with Ag_29_-PPh_3_, the Ag_29_-PPh_2_py nanocluster contained retained S-Adm ligands, while the surface PPh_3_ ligands were altered to PPh_2_py, and several NO_3_ ligands were arranged on the nanocluster surface. Color legends: light blue sphere, Ag; red sphere, S; magenta sphere, P; blue sphere, N; green sphere, O. For clarity, all C and H atoms are omitted. Of note, the structure of Ag_29_-PPh_3_ is proposed in this figure.

Ag_29_-PPh_3_ was prepared by a literature method.^46^ Although the Ag_29_-PPh_3_ nanocluster was uncrystallizable because of its weak stability, several of its alloyed derivatives have been structurally determined, including Pt_1_Ag_28_-PPh_3_, Au_1_Ag_28_(S-Adm)_18_(PPh_3_)_4_, and Pt_1_Ag_12_Cu_16_(S-Adm)_18_(PPh_3_)_4_.^44–46^ In this context, alloying has been used as an efficient approach to improve the stability of the M_29_ framework.^46^Fig. 1 depicts the proposed structure of Ag_29_-PPh_3_. Of note, the Ag_13_ kernel in Ag_29_-PPh_3_ might follow a FCC (face-centered cubic) configuration for two reasons: (i) the consistent FCC configuration of the M_13_ kernel in PPh_3_ and S-Adm co-stabilized M_29_ nanoclusters,^44–46^ and (ii) the different absorption profiles of Ag_29_-PPh_3_ and Ag_29_-PPh_2_py (discussed below). However, such a verification calls for more experimental efforts.

At the same time, we unremittingly made efforts to stabilize the homo-silver Ag_29_ and determine its atomically precise structure. Considering that (i) the unchanging S-Adm ligand could retain the basic framework of the Ag_29_ nanocluster^47,48^ and (ii) the introduction of N-coordination sites in original ligands would generate new N–metal interactions that might enhance the structural robustness,^49–52^ we were motivated to substitute the PPh_3_ ligand with PPh_2_py while retaining the S-Adm ligand in the nanocluster synthesis. A new Ag_29_ nanocluster, formulated as Ag_29_(S-Adm)_15_(NO_3_)_3_(PPh_2_py)_4_ (Ag_29_-PPh_2_py), was synthesized and further structurally determined owing to its high stability (Fig. 1 and S1†).

Compared with Ag_29_-PPh_3_, Ag_29_-PPh_2_py contained three fewer S-Adm ligands and three more NO_3_ ligands, and the number of the phosphine ligands retained was four (Fig. 1). Because of the interactions between N (in PPh_2_py) and Ag (in the cluster), the surface structure of Ag_29_-PPh_2_py displayed more obvious distortion relative to Ag_29_-PPh_3_ (Fig. 1 and S2†). Besides, three NO_3_ ligands were observed on the nanocluster surface *via* Ag–O interactions. For the three O atoms in each NO_3_, the two inward O linked to two Ag atoms or one Ag atom, while the outward O was naked (Fig. 1 and S2†). The presence of NO_3_ in the cluster system has been verified by IR measurement (Fig. S3†). ESI-MS measurement was performed to validate the molecular composition and determine the valence state of the nanocluster. As shown in Fig. S4,† the experimental mass signals at 2292.30 and 2271.64 Da matched well with the theoretical results of [Ag_29_(S-Adm)_15_(NO_3_)_3_(PPh_2_py)_4_]^3+^ and [Ag_29_(S-Adm)_15_(NO_3_)_2_(PPh_2_py)_4_]^3+^, respectively. In this context, the NO_3_ ligand on the nanocluster surface was more prone to be dissociated relative to S-Adm and PPh_2_py ligands. Besides, the “+3” valence state of Ag_29_-PPh_2_py was tallied with the presence of 3SbF_6_^−^ counterions with an Ag_29_ cluster molecule in the crystal lattice (Fig. S1†). According to the valence state of the Ag_29_-PPh_2_py nanocluster, its nominal electron count was determined to be 8,^53^*i.e.*, 29(Ag) − 15(SR) − 3(NO_3_) − 3(charge) = 8*e*, the same as that of Ag_29_-PPh_3_.

**Fig. 2:**
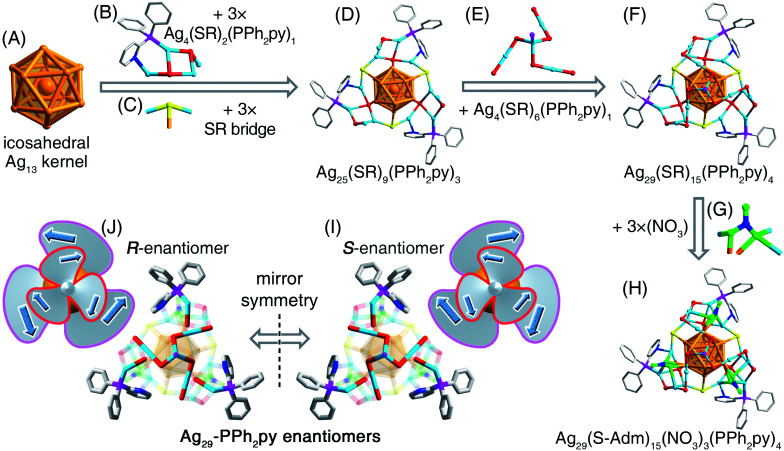
Structural anatomy of the Ag_29_-PPh_2_py nanocluster. (A) The icosahedral Ag_13_ kernel. (B) and (C) The Ag_4_(S-Adm)_2_(PPh_2_py)_1_ surface and S-Adm bridge-like units. (D) The Ag_25_(S-Adm)_9_(PPh_2_py)_3_ structure. (E) The Ag_4_(S-Adm)_6_(PPh_2_py)_1_ surface unit. (F) Ag_29_(S-Adm)_15_(PPh_3_py)_4_ structure. (G) The surface NO_3_ ligand. (H) Overall structure of the Ag_29_-PPh_2_py nanocluster. (J) and (I) The Ag_29_-PPh_2_py nanocluster enantiomers. Color legends: orange sphere, kernel Ag; light blue sphere, surface Ag; red/yellow sphere, S; magenta sphere, P; blue sphere, N; green sphere, O. For clarity, all H atoms and several C atoms are omitted.

Structurally, the Ag_29_-PPh_2_py nanocluster contains an icosahedral Ag_13_ kernel (Fig. 2A). Of note, for other structurally determined M_29_(S-Adm)_18_(PR_3_)_4_ nanoclusters, their Ag_13_ kernels follow a FCC configuration.^46^ The difference between these two kernel configurations originates from their distinguishable surface environments *via* a “surface-kernel structure transfer effect”. The Ag_13_ kernel of Ag_29_-PPh_2_py is first wrapped by three same Ag_4_(S-Adm)_2_(PPh_2_py)_1_ motif structures that are further fixed by three S-Adm bridges (Fig. 2B and C), giving rise to an Ag_25_(S-Adm)_9_(PPh_2_py)_3_ structure (Fig. 2D). Such three Ag_4_(S-Adm)_2_(PPh_2_py)_1_ motifs or three S-Adm bridges are in *C*_3_ axial symmetry. Besides, an Ag_4_(S-Adm)_6_(PPh_2_py)_1_ surface unit caps the Ag_25_(S-Adm)_9_(PPh_2_py)_3_ structure to present an Ag_29_(S-Adm)_15_(PPh_3_py)_4_ structure (Fig. 2E and F). In this context, the four PPh_2_py ligands follow different bonding modes in the nanocluster framework: three PPh_2_py are dually bonded onto the nanocluster *via* both Ag–P and Ag–N interactions, while the remaining one is singly bonded onto the nanocluster vertex *via* the Ag–P interaction (Fig. S2†). Of note, the Ag_29_(S-Adm)_15_(PPh_3_py)_4_ structure is still bare to a certain extent, and three NO_3_ ligands, which originated from the AgNO_3_ reactant, are further anchored onto the nanocluster surface (Fig. 2G), making the Ag_29_ kernel fully protected and yielding the overall structure of Ag_29_-PPh_2_py (Fig. 2H). The complete structure of Ag_29_-PPh_2_py follows a *C*_3_ axial symmetry, and the axis of the symmetry passes through the vertex P and the innermost Ag atoms (Fig. S5†).

In the crystal lattice of Ag_29_-PPh_2_py, two nanocluster enantiomers were observed, labeled as the *R*-nanocluster enantiomer and *S*-nanocluster enantiomer in Fig. 2I and J. Each type of enantiomer displayed a bilayer rotation: (i) for the *S*-nanocluster enantiomer, the inner-layer (*i.e.*, the Ag_4_(S-Adm)_6_(PPh_2_py)_1_) was counterclockwise while the outer-layer (*i.e.*, assembly of three surface Ag_1_(S-Adm)_1_(PPh_2_py)_1_) was clockwise (Fig. 2I); (ii) for the *R*-nanocluster enantiomer, the rotations of the inner-layer and outer-layer were opposite to those of the *S*-nanocluster enantiomer (Fig. 2J). Since the quantities of *R*- and *S*-nanocluster enantiomers are the same in the crystal lattice, the nanocluster samples were racemic.

The Ag_29_-PPh_3_ and Ag_29_-PPh_2_py nanoclusters with distinguishable kernel structures and surface environments exhibited different optical absorptions. The CH_2_Cl_2_ solution of Ag_29_-PPh_3_ showed an intense absorption at 413 nm and a shoulder band at 506 nm (Fig. S6,† black line). By comparison, the CH_2_Cl_2_ solution of Ag_29_-PPh_3_ showed several apparent UV-vis signals at 401, 438, and 530 nm (Fig. S6,† red line). The difference in optical absorptions of these two Ag_29_ nanoclusters suggested their distinct electronic structures.^54,55^ The photoluminescence properties of Ag_29_-PPh_3_ and Ag_29_-PPh_2_py nanoclusters were further compared. As shown in Fig. S7,† the CH_2_Cl_2_ solution of Ag_29_-PPh_3_ was red emissive with an intense signal at 622 nm. By comparison, the Ag_29_-PPh_2_py was non-emissive in the solution state. The different photophysical properties originated from their distinct electronic structures.^54,55^

**Fig. 3:**
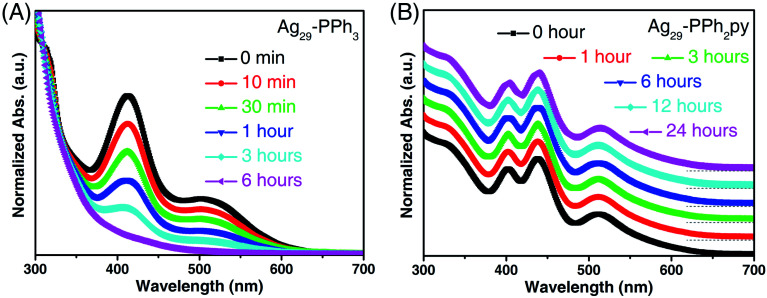
Stability of different Ag_29_ nanoclusters. (A) Time-dependent optical absorptions of Ag_29_-PPh_3_ in CH_2_Cl_2_ in air. (B) Time-dependent optical absorptions of Ag_29_-PPh_2_py in CH_2_Cl_2_ in air.

The thermal stability of these two Ag_29_ nanoclusters was then compared in air. As shown in Fig. 3A, the characteristic optical peaks of Ag_29_-PPh_3_ continuously decreased in the first three hours and completely disappeared within six hours, demonstrating the decomposition of the nanoclusters. In this context, the Ag_29_-PPh_3_ nanocluster was unstable. In vivid contrast, the optical absorptions of Ag_29_-PPh_2_py remained unchanged for 24 hours (Fig. 3B), which suggested the high robustness of this nanocluster. Besides, the difference in stability was primarily responsible for the crystallographic discrepancy of these two Ag_29_ nanoclusters: the Ag_29_-PPh_3_ nanocluster was uncrystallizable, whereas the crystal structure of Ag_29_-PPh_2_py was successfully determined.

**Fig. 4:**
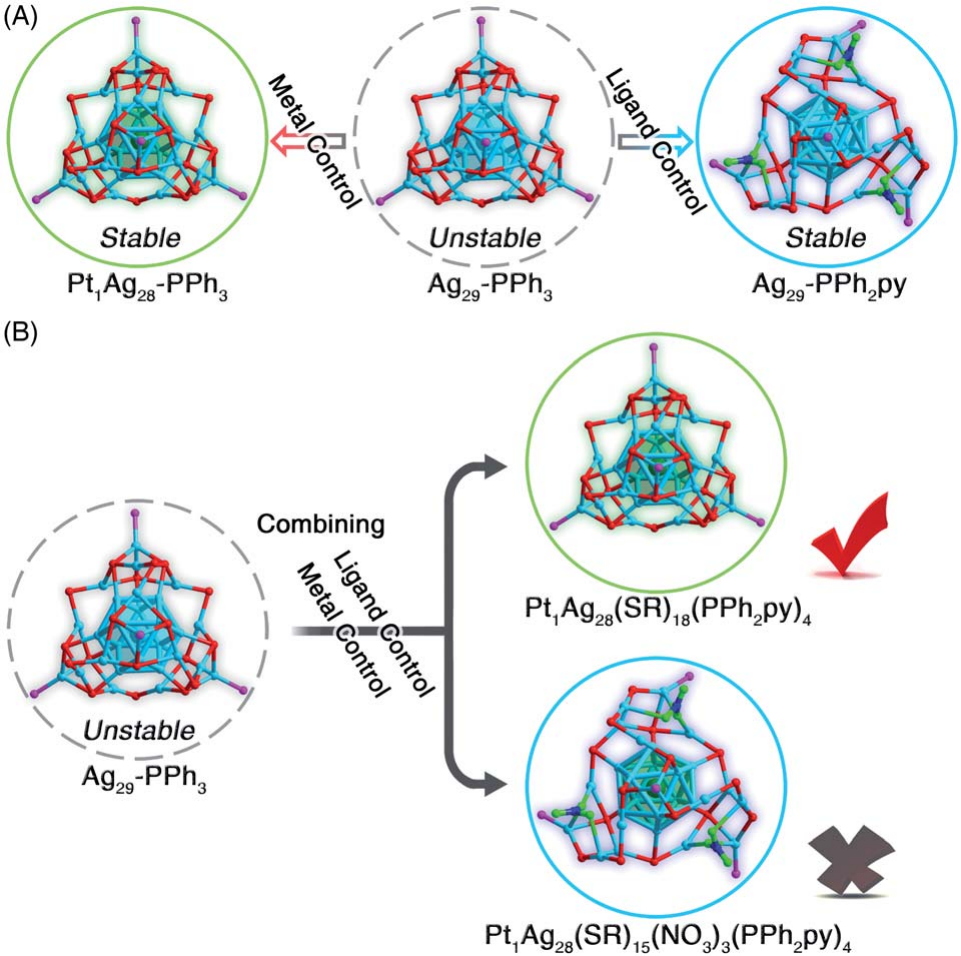
Metal control *versus* ligand control on the Ag_29_ nanocluster template. (A) From unstable Ag_29_-PPh_3_ to stable Pt_1_Ag_28_-PPh_3_*via* metal control, or from unstable Ag_29_-PPh_3_ to stable Ag_29_-PPh_2_py*via* ligand control. (B) From unstable Ag_29_-PPh_3_ to stable Pt_1_Ag_28_-PPh_2_py*via* both metal control and ligand control. Color legends: light blue sphere, Ag; dark green sphere, Pt; red sphere, S; magenta sphere, P; blue sphere, N; green sphere, O. For clarity, all C and H atoms are omitted.

Collectively, as depicted in Fig. 4A, two approaches have been presented to endow the unstable Ag_29_-PPh_3_ nanocluster with enhanced stability: (i) the metal control approach (*e.g.*, from unstable Ag_29_-PPh_3_ to stable Pt_1_Ag_28_-PPh_3_),^46^ and (ii) the ligand control approach (*i.e.*, from unstable Ag_29_-PPh_3_ to stable Ag_29_-PPh_2_py). These two disparately stabilizing approaches raised an interesting question: which type of the Pt_1_Ag_28_ nanocluster would be generated when the metal control and the ligand control were performed simultaneously in the synthesis (Fig. 4B)?

As inspired by the aforementioned results, two types of Pt_1_Ag_28_ nanoclusters with different surface environments might be generated (Fig. 4B): Pt_1_Ag_28_(S-Adm)_18_(PPh_2_py)_4_ with a maintained framework or Pt_1_Ag_28_(S-Adm)_15_(NO_3_)_3_(PPh_2_py)_4_ with a twisted framework. After the crystallographic analysis, we determined its structure as the framework-retained Pt_1_Ag_28_(S-Adm)_18_(PPh_2_py)_4_ (Pt_1_Ag_28_-PPh_2_py for short). The structure of Pt_1_Ag_28_-PPh_2_py was almost the same as that of Pt_1_Ag_28_-PPh_3_ (Fig. S8†).^44,46^ Although the four PPh_2_py ligands in Pt_1_Ag_28_-PPh_2_py exposed N coordination sites, these N sites remained uncoordinated in the nanocluster formation (Fig. S8†). Consequently, in the competition between metal control and ligand control in this nanocluster system, the metal control seized a dominant position (Fig. 4B). In other words, when the Pt heteroatom was introduced into the innermost region of the nanocluster, the M_29_ structure was robust enough to hinder the formation of surface Ag–N interactions, which resulted in a retained cluster framework without any distortion. Besides, in the previously reported intercluster transformation from Pt_1_Ag_28_-PPh_3_ into Pt_1_Ag_28_(BDT)_12_(PPh_3_)_4_ (BDT = 1,3-benzenedithiolate), the presence of BDT afforded the kernel transformation from FCC into icosahedron.^56^ In this context, for the Pt_1_Ag_28_ cluster template, the bidentate thiolate ligand (*i.e.*, BDT) showed enhanced ability for directing the nanocluster configuration relative to the bidentate phosphine ligand (*i.e.*, PPh_2_py).

**Fig. 5:**
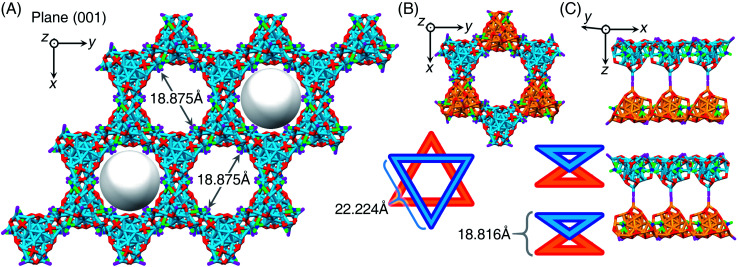
Packing of Ag_29_-PPh_2_py in the supracrystal lattice. (A) Crystalline packing of Ag_29_-PPh_2_py, viewed from the (001) plane. (B) Vertical and (C) lateral views of the aggregation pattern of Ag_29_-PPh_2_py molecules in the supracrystal lattice. Color legends: light blue sphere, Ag in the *S*-nanocluster enantiomer; orange sphere, Ag in the *R*-nanocluster enantiomer; red sphere, S; magenta sphere, P; blue sphere, N; green sphere, O. For clarity, all C and H atoms are omitted.

The Ag_29_-PPh_2_py nanocluster molecules followed a crystallographic pattern of “lamellar eutectic” between *R*-nanocluster and *S*-nanocluster enantiomers, viewed from both *x* and *y* axes (Fig. S9A–C†). The interlayer distance along the *z* axis was determined to be 34.064 Å (from cluster kernel to cluster kernel, as shown in Fig. S9B†). Significantly, the supracrystal lattice of Ag_29_-PPh_2_py showed several intercluster channels with the same diameter of 18.875 Å from the (001) crystalline plane (Fig. 5A and S9D†), which was reminiscent of the behavior of MOFs (metal–organic frameworks).^57,58^ However, the channel diameter should be remarkably less than 18.875 Å due to the presence of carbon tails from peripheral ligands of nanoclusters (Fig. S10†). The intercluster channel was constructed by symmetrically assembling six cluster molecules into a hexagon, where three molecules were *R*-nanocluster enantiomers (marked in orange in Fig. 5B), while the other three were *S*-nanocluster enantiomers (marked in blue in Fig. 5B). Specifically, the intercluster hexagon was composed of two cluster-based triangles in parallel planes in opposite directions, and each triangle contained three cluster molecules in the same enantiomeric configuration (Fig. 5B and C). The intermolecular distance of the cluster-based triangle was 22.224 Å, and the interlayer distance between two adjacent triangles was 18.816 Å (Fig. 5B and C). Furthermore, the arrangement of SbF_6_^−^ counterions in the supracrystal lattice was analyzed. As shown in Fig. S11,† 2/3 of SbF_6_^−^ counterions were uniformly organized in the intercluster channels while the others were packed along the *C*_3_ axis of symmetry of Ag_29_-PPh_2_py nanoclusters. Of note, such a hexagon-like crystallographic packing of Ag_29_-PPh_2_py cluster molecules in the supracrystal lattice was unique, which has yet been detected in other M_29_ nanocluster crystals.^44–46,48,59,60^ For example, for the crystal lattice of Pt_1_Ag_28_-PPh_2_py, the nanocluster molecules were packed in a layered assembly mode from the *x* axis, *y* axis, or *z* axis, and no intercluster channel was detected (Fig. S12†). In this context, such unique intercluster channels may render the Pt_1_Ag_28_-PPh_2_py crystals potential nanomaterials for gas adsorption-related applications.^61–65^

## 4 Conclusions

In summary, a strategy termed “surface environment complication” has been exploited to render unstable Ag_29_ highly robust. The surface structure of unstable Ag_29_(S-Adm)_18_(PPh_3_)_4_ underwent directional distortion due to the generation of Ag–N interactions by substituting the monodentate PPh_3_ ligand with bidentate PPh_2_py. Besides, three NO_3_ ligands were anchored onto the nanocluster surface to entirely protect the Ag_29_ kernel, yielding a new Ag_29_(S-Adm)_15_(NO_3_)_3_(PPh_2_py)_4_ nanocluster with high robustness. Owing to its enhanced stability, the Ag_29_(S-Adm)_15_(NO_3_)_3_(PPh_2_py)_4_ nanocluster was crystallizable, and its atomically precise structure was successfully determined. On the supramolecular level, the Ag_29_(S-Adm)_15_(NO_3_)_3_(PPh_2_py)_4_ nanocluster molecules followed a unique crystallographic packing mode and displayed several intercluster channels. This study thus presented a novel strategy for tailoring the surface environment of metal nanoclusters, and also provided fundamental insights into the controllable synthesis of highly robust silver nanoclusters. Future work will focus on promoting this strategy to other ligand-protected metal nanoclusters.

## Data availability

All the data supporting this article have been included in the main text and the ESI.†

## Author contributions

C. X. and Q. Y. carried out the experiments and analyzed the data. X. W., H. L. and H. S. assisted in the analysis. X. K. and M. Z. designed the project, analyzed the data, and wrote the manuscript.

## Conflicts of interest

There are no conflicts to declare.

## Supplementary Material

SC-013-D1SC06002C-s001

SC-013-D1SC06002C-s002
