# Supplementary material for: Surface environment complication makes Ag29 nanoclusters more robust and leads to their unique packing in the supracrystal lattice
Source: Chem Sci. 2022 Jan 3;13(5):1382–9. doi: 10.1039/d1sc06002c (PMC8809389; doi:10.1039/d1sc06002c)
Supplement: SC-013-D1SC06002C-s001 [file SC-013-D1SC06002C-s001.pdf]

Electronic Supplementary Material (ESI) for Chemical Science.
This journal is © The Royal Society of Chemistry 2022

Supporting Information

Surface environment complication makes Ag_{29} nanoclusters more robust and leads to their unique packing in the supracrystal lattice

Chao Xu,[#] Qianqin Yuan,[#] Xiao Wei, Hao Li, Honglei Shen, Xi Kang,* Manzhou Zhu*

Department of Chemistry and Centre for Atomic Engineering of Advanced Materials, Key Laboratory of Structure and Functional Regulation of Hybrid Materials of Ministry of Education, Institutes of Physical Science and Information Technology and Anhui Province Key Laboratory of Chemistry for Inorganic/Organic Hybrid Functionalized Materials, Anhui University, Hefei, Anhui 230601, China.

#C.X. and Q.Y. contributed equally to this work.

*E-mails of corresponding authors: kangxi_chem@ahu.edu.cn (X.K.); zmz@ahu.edu.cn (M.Z.).

Notes: The authors declare no competing financial interest.

This Supporting Information includes:

Figures S1-S12

Tables S1-S2

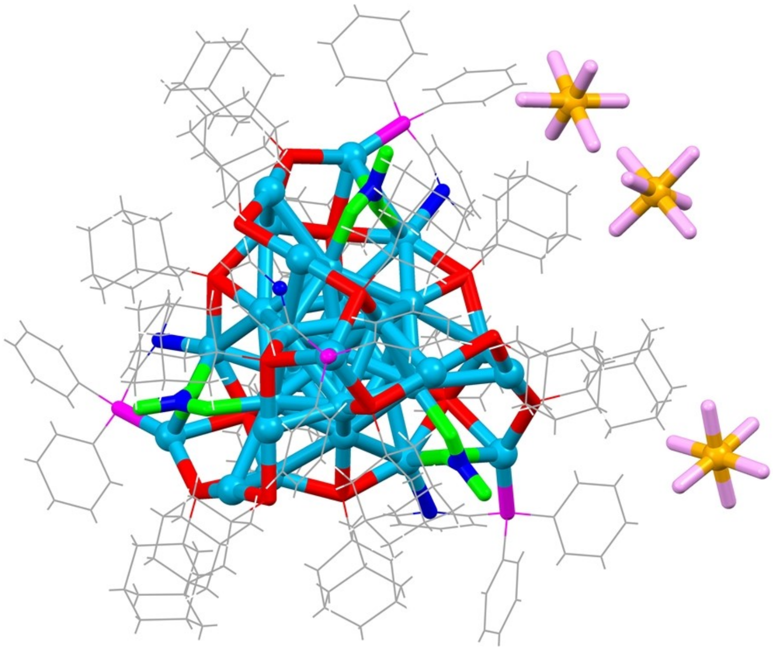

Fig. S1 The overall structure of the $[Ag_{29}(S\text{-}Adm)_{15}(NO_3)_3(PPh_2py)_4](SbF_6)_3$ nanocluster. Color legends: light blue sphere, Ag; red sphere, S; magenta sphere, P; blue sphere, N; green sphere, O; orange sphere, Sb; pink sphere, F; grey sphere, C; white sphere, H.

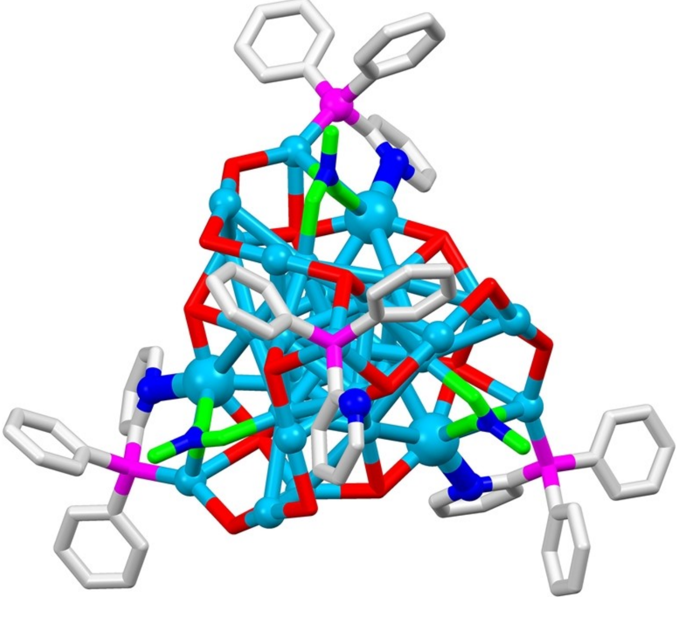

Fig. S2 Binding modes of the four PPh_2py and three NO_3 ligands on the nanocluster surface. Three PPh_2py ligands were anchored onto the nanocluster surface via Ag-P and Ag-N interactions, while the remaining PPh_2py ligand was anchored onto the nanocluster surface via only Ag-P interaction. The NO_3 ligand was anchored onto the nanocluster surface via Ag-O interactions. Color legends: light blue sphere, Ag; red sphere, S; magenta sphere, P; blue sphere, N; green sphere, O; grey sphere, C. For clarity, all H atoms and several C atoms are omitted.

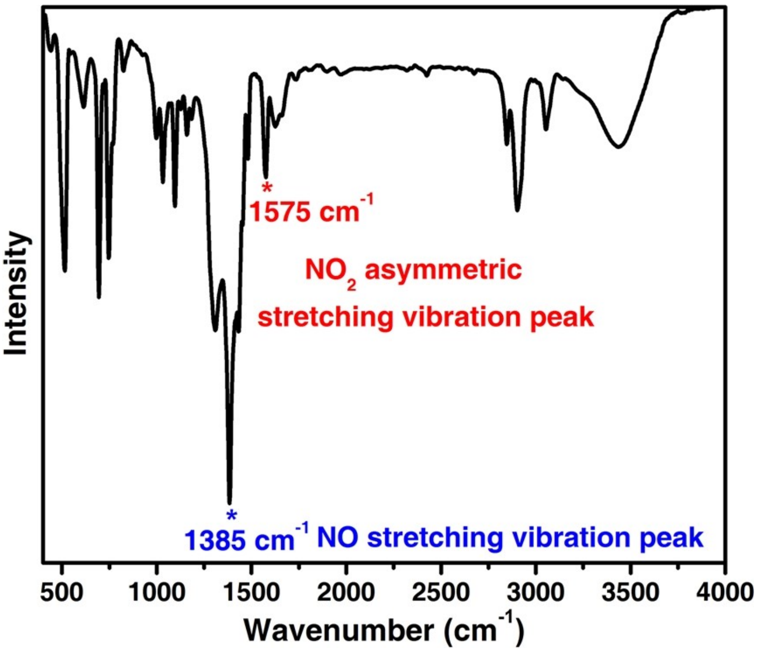

Fig. S3 IR result of the **Ag_{29}-PPh_2py** nanocluster. Two IR signals were observed, corresponding to NO stretching vibration peak (1385 cm^{-1}, highlighted in blue) and NO_2 asymmetric stretching vibration peak (1575 cm^{-1}, highlighted in red), which demonstrated the presence of NO_3 ligands on the nanocluster surface.

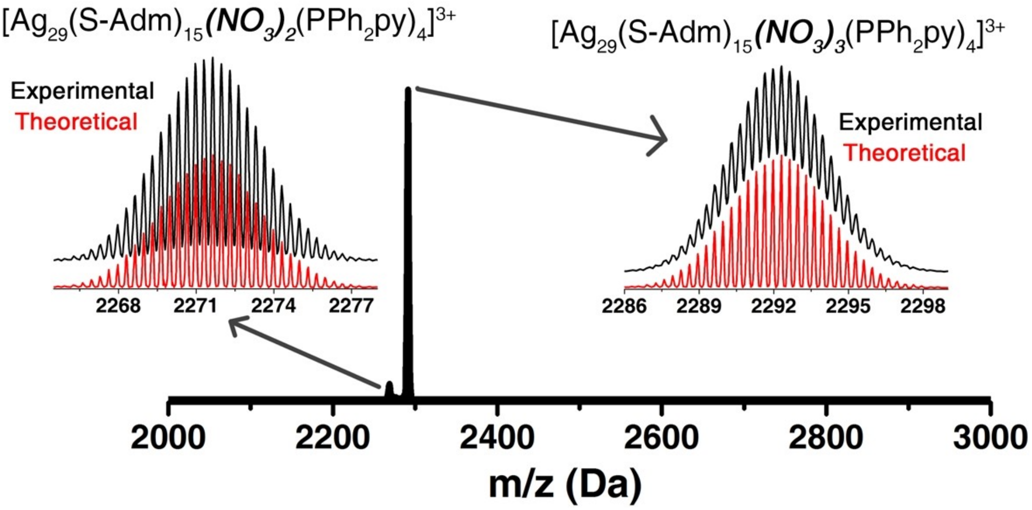

Fig. S4 ESI-MS result of the **Ag_{29}-PPh_2py** nanocluster in a positive mode in a mass range from 2000 to 3000 Da. Two mass signals were observed, corresponding to $[Ag_{29}(S\text{-}Adm)_{15}(NO_3)_3(PPh_2py)_4]^{3+}$ and $[Ag_{29}(S\text{-}Adm)_{15}(NO_3)_2(PPh_2py)_4]^{3+}$, respectively.

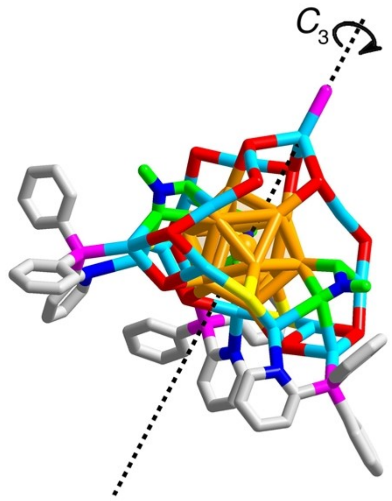

Fig. S5 The C_3 axis of symmetry of the $Ag_{29}(S\text{-}Adm)_{15}(NO_3)_3(PPh_2py)_4$, which passed through the vertex P and the innermost Ag atoms.

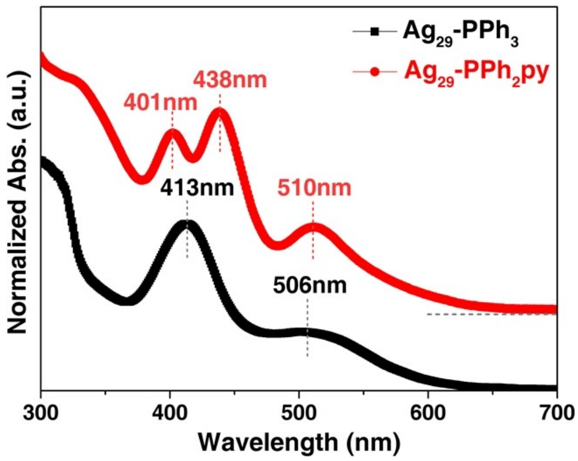

Fig. S6 Comparison of UV-vis spectra between **Ag_{29}-PPh_3** (black line) and **Ag_{29}-PPh_2py** (red line).

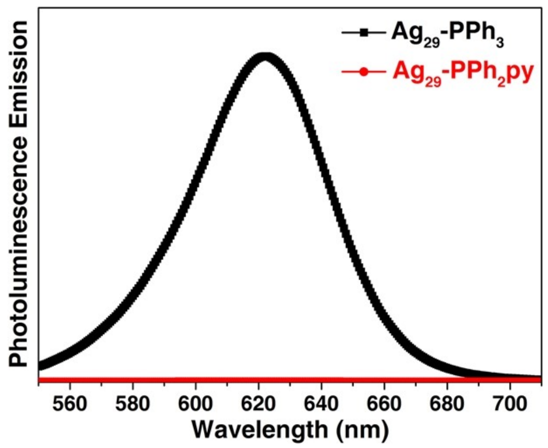

Fig. S7 Comparison of photoluminescent emissions between **Ag_{29}-PPh_3** (black line) and **Ag_{29}-PPh_2py** (red line) nanoclusters. Nanoclusters were dissolved in CH_2Cl_2 with the same OD of 0.1.

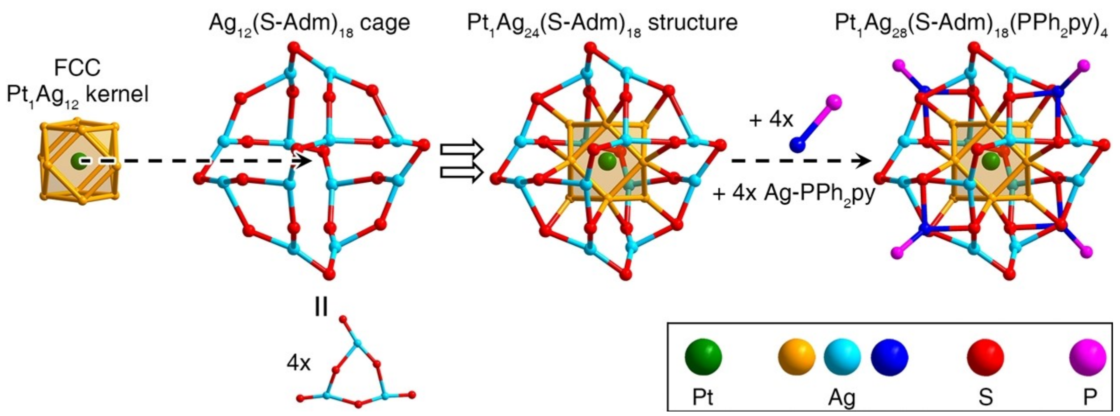

Fig. S8 Structural anatomy of the $Pt_1Ag_{28}(S\text{-}Adm)_{18}(PPh_2py)_4$ nanocluster. The nanocluster comprised a FCC Pt_1Ag_{12} kernel, an $Ag_{12}(S\text{-}Adm)_{18}$ cage, and four Ag-PPh_2py vertex units. The overall configuration of $Pt_1Ag_{28}(S\text{-}Adm)_{18}(PPh_2py)_4$ was the same as that of $Pt_1Ag_{28}(S\text{-}Adm)_{18}(PPh_3)_4$. Color legends: orange/light blue/dark blue sphere, Ag; red sphere, S; magenta sphere, P. For clarity, all C, N, and H atoms are omitted.

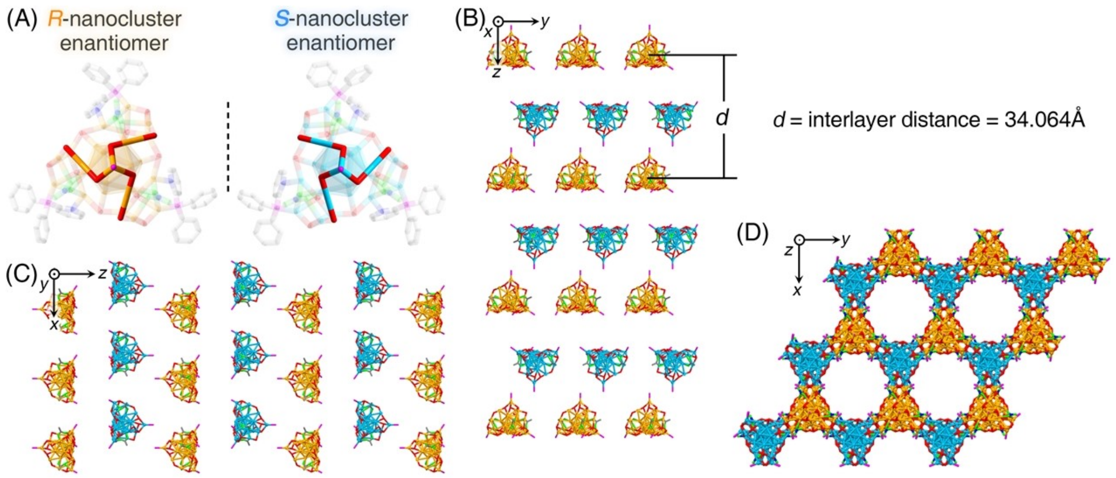

Fig. S9 (A) Structures of *R*-nanocluster and *S*-nanocluster enantiomers. (B-D) Packing of **Ag_{29}-PPh_2py** nanoclusters in the crystal lattice: view from the *x* axis (B), *y* axis (C) and *z* axis (D). As depicted in (B), the interlayer distance was 34.064 Å.

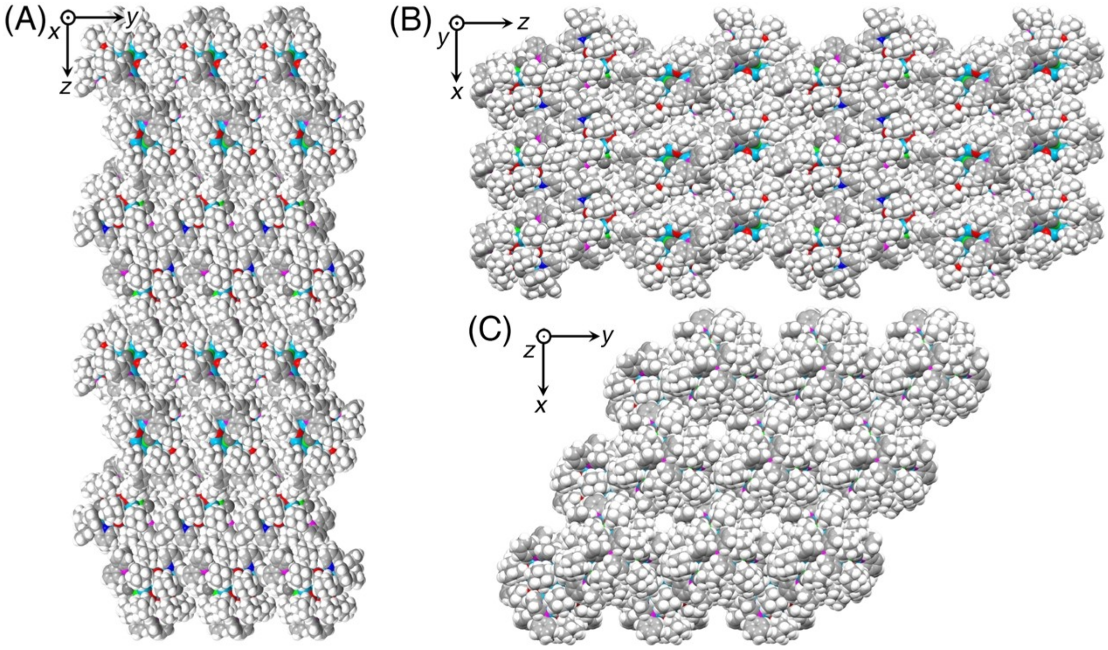

Fig. S10 Packing of **Ag_{29}-PPh_2py** nanoclusters (in a Spacefill mode) in the crystal lattice: view from the *x* axis (A), *y* axis (B) and *z* axis (C).

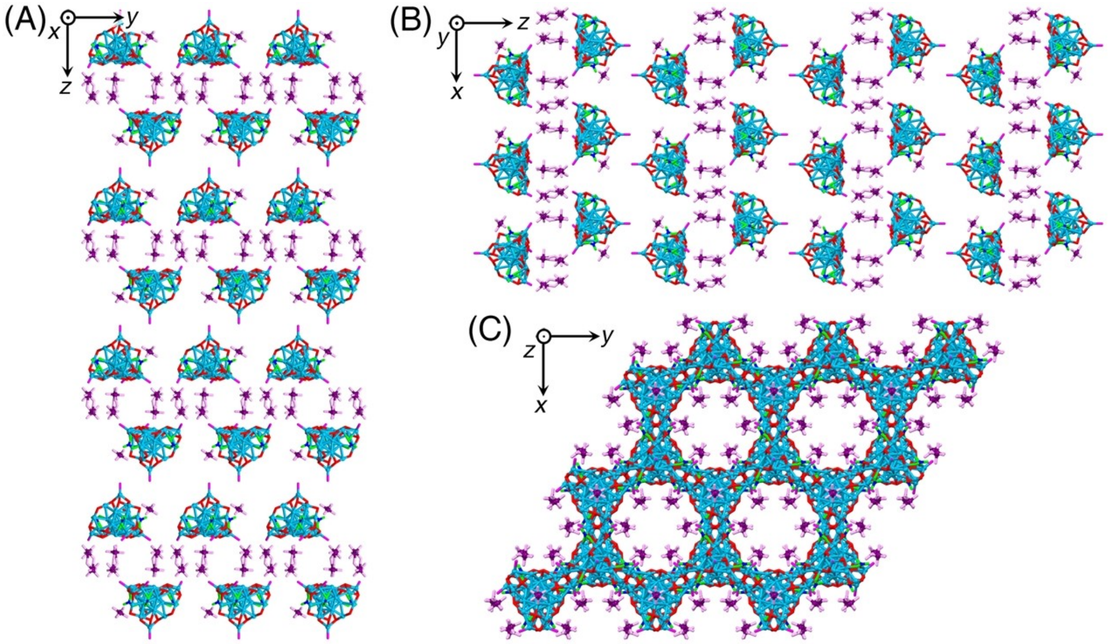

Fig. S11 Packing of **Ag_{29}-PPh_2py** nanoclusters and SbF_6^- counterions in the crystal lattice: view from the *x* axis (A), *y* axis (B) and *z* axis (C).

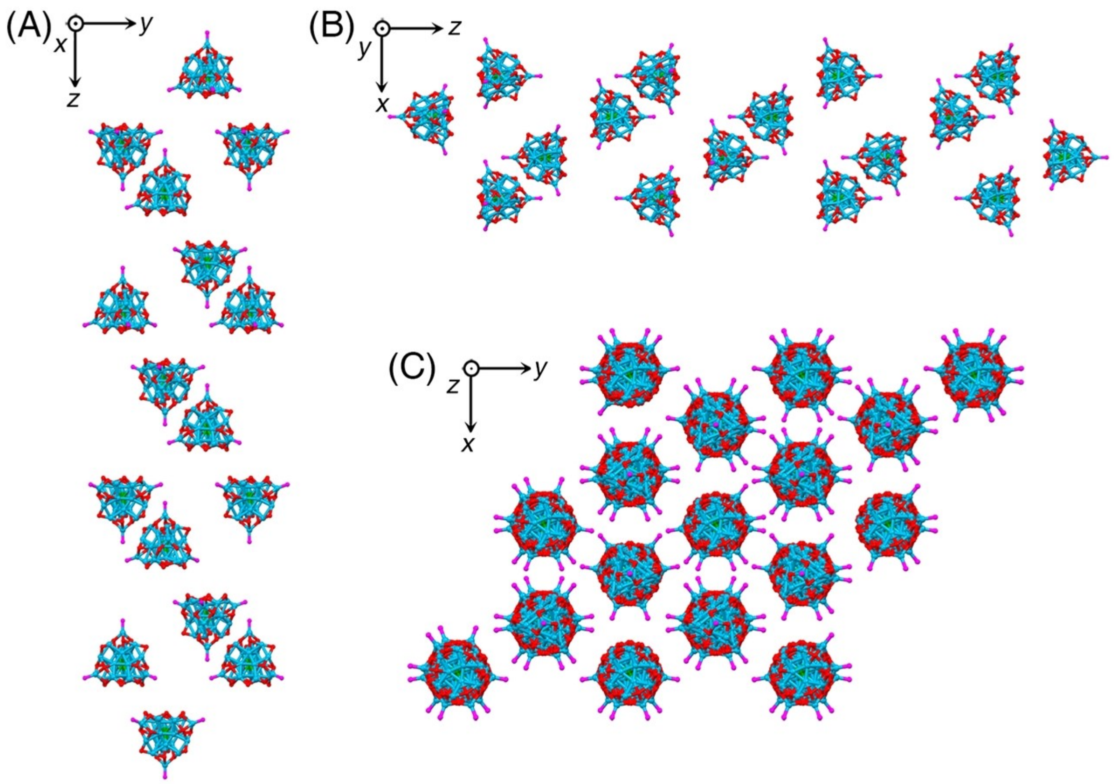

Fig. S12 Packing of **Pt_1Ag_{28}-PPh_2py** nanoclusters in the crystal lattice: view from the *x* axis (A), *y* axis (B) and *z* axis (C).

Table S1. Crystal data and structure refinement for the **Ag_{29}-PPh_2py**.

Crystal system	trigonal
Space group	*P* -3 *c* 1
a/Å	22.224(4)
b/Å	22.224(4)
c/Å	68.128(12)
α/°	90
β/°	90
γ/°	120
Volume/$Å^3$	29141(12)
Z	4
ρcalcg/cm^3	1.701
μ/mm^{-1}	2.307
F(000)	14534
Radiation	MoKα (λ = 0.71073)
Index ranges	-28 ≤ h ≤ 28, -28 ≤ k ≤ 28, -78 ≤ l ≤ 84
Final R indexes [I>=2σ (I)]	R1 = 0.1337, wR2 = 0.3296
Final R indexes [all data]	R1 = 0.1651, wR2 = 0.3092

Table S2. Crystal data and structure refinement for the **Pt_1Ag_{28}-PPh_2py**.

Crystal system	trigonal
Space group	R -3 c
a/Å	29.410(8)
b/Å	29.410(8)
c/Å	141.76(4)
α/°	90
β/°	90
γ/°	120
Volume/Å^3	106187(63)
Z	12
ρcalcg/cm^3	1.364
μ/mm^{-1}	14.251
F(000)	42936
Radiation	CuKα (λ = 1.54186)
Index ranges	-28 ≤ h ≤ 33, -33 ≤ k ≤ 13, -151 ≤ l ≤ 162
Final R indexes [I>=2σ (I)]	R1 = 0.0764, wR2 = 0.2450
Final R indexes [all data]	R1 = 0.1103, wR2 = 0.2112
